# Age estimation by evaluation of pulp chamber to crown volume of central incisor and first molar of maxilla, using Cone-Beam CT

**DOI:** 10.1016/j.heliyon.2024.e40022

**Published:** 2024-11-04

**Authors:** Atie Safaei, Ali Bagherpour, Shahrokh Naseri, Mahsa Etemadi, Hossein Khoshkhou

**Affiliations:** a Department of Oral and Maxillofacial Radiology, School of Dentistry, Mashhad University of Medical Sciences, Mashhad, Iran; b Department of Medical Physics, Faculty of Medicine, Mashhad University of Medical Sciences, Mashhad, Iran; c Department of Periodontics, School of Dentistry, Tehran University of Medical Sciences, Tehran, Iran; d Department of Periodontics, School of Dentistry, Mashhad University of Medical Sciences, Mashhad, Iran

**Keywords:** Cone-beam computed tomography, Dental pulp cavity, Age estimation, Single upper central incisor, Maxillary molar teeth

## Abstract

**Objectives:**

This study examines the relationship between chronological age and the ratio of pulp chamber volume to crown volume in maxillary central incisors and first molars. Cone-beam computed tomography (CBCT) is used to measure the pulp chamber size, which decreases following the eruption of the tooth.

**Study design:**

The study focused on age estimation using cone beam computed tomography (CBCT) images of 156 patients from Mashhad Dental School (67 maxillary central incisors and 42 maxillary first molars). We stratified the age range into three groups: 18–27 years, 28–37 years, and 38–49 years. The study used Planmeca ProMax 3D and ITK-SNAP 3.4.0 software for segmentation and volume quantification. The data was analyzed using PASW Statistics 18 software, with descriptive statistics and linear regression analysis for age estimation.

**Results:**

This study created a mathematical model for calculating chronological age, taking into account factors such as pulp-to-crown volume ratio, gender, and tooth type. Results showed no significant difference in mean age between male and female subjects, and the average pulp-to-crown volume ratio remained consistent across genders and tooth types.

**Conclusion:**

The study uses CBCT to estimate Iranian population age using maxillary central incisors and first molars. The pulp chamber to crown volume ratio is reliable for 28–37 years, but accuracy decreases with age. Larger sample sizes and other teeth could improve estimation.

## 1 Introduction

Age estimation holds great importance in the field of forensic science, as it serves as a crucial method for evaluating the age of both live humans and cadavers. The significance of teeth stems from their extraordinary ability to withstand damage, thanks to their great chemical and physical strength [1,2]. Unlike most other tissues in the human body, teeth are not significantly influenced by environmental and living factors.

Consequently, numerous sophisticated methods have been developed for age estimation, with a focus on various tooth characteristics, including growth patterns and degree of calcification within the oral cavity [[3], [4], [5], [6]].

The practice of age estimation carries a multifaceted purpose, extending to individual identification as well as the elucidation of historical mortality trends [4]. However, it is essential to acknowledge that age estimation, while invaluable, is not an exact science and can exhibit variations of approximately 4.12 years in individual cases. Nevertheless, it is an invaluable guiding tool in the field of forensic medicine that offers critical insights into the lives and demise of individuals [7].

In a pioneering study in 1995, Kvaal et al. introduced a novel method centered on the evaluation of secondary dentin secretion through radiography. This groundbreaking approach has enabled precise measurement of dental pulp and its intricate relationship with age, representing a significant advancement in the field. Subsequently, a plethora of studies have consistently highlighted age using through dental radiographic measurements as the most widely adopted and reliable technique for determining an individual's age, underscoring its pivotal role in forensic investigations [8].

Cameriere et al. conducted a comprehensive study that delved into the nuanced intricacies of age estimation. Their research, which involved assessing the tooth-to-pulp ratio in different dental elements, has added a substantial layer of sophistication to this field of study [7]. While age estimation in individuals up to 24 years of age predominantly relies on the orderly progression and developmental stages of tooth growth, a distinct paradigm emerges when evaluating adults, where the focus shifts towards the assessment of age after the maturation of the third molar bud [6,9,10]. The introduction of Cone-Beam Computed Tomography (CBCT) has brought about a new era in age assessment in the field of forensic dentistry. CBCT technology enables the acquisition of three-dimensional tooth structures with exceptional accuracy, resolving the problems of picture overlap and distortion. This technological development has completely changed the field and revealed important new information on the structural alterations in teeth and how closely they correlate with age. This is especially important for determining the postmortem age, as CBCT has shown to be an invaluable tool in this regard [9].

With an emphasis on maxillary central incisors and first molars, this study focused on using the huge potential of CBCT images to develop a reliable model for age assessment in the Iranian population.

## 2 Materials and methods

This study was approved by the Ethics Committee of the School of Dentistry with reference number IR.MUMS.DENTISTRY.REC.1398.027.

**Fig. 1:**
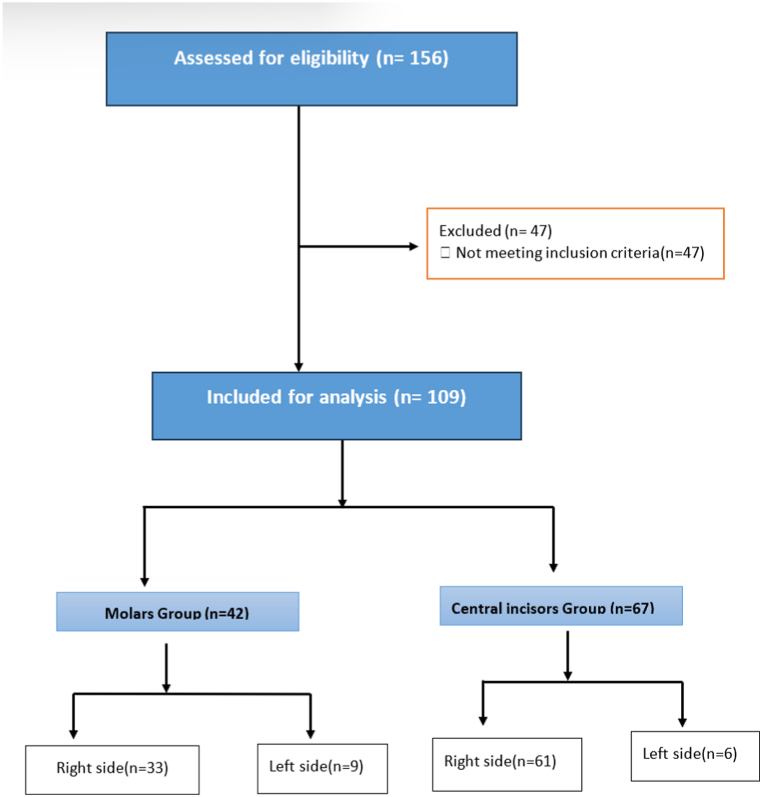
Flowchart of the number of patients participating in the study.

This study was performed on CBCT images of 156 (recorded by the Planmeca ProMax 3D Classic device, Finland, voxel size = .15 mm^3^) patients referred to Mashhad dental school. Only 109 out of 156 CBCT images met the inclusion criteria (Fig. 1). Descriptive findings are presented in Table 1.

**Table 1:** Descriptive data of the subjects.

Gender		Tooth		Mean Age ± S.d∗	Minimm Age	Maximm Age	Total Number
		Central Incisor (%)	Maxillary First Molar (%)				
Male	Count 28(63.6)		16(36.4)	31.931 ± 7.425	18.00	49.00	44
Female	39(60)		26(40)	32.984 ± 7.199	20.00	49.00	65
Total		67(61.5)	42(38.5)	32.559 ± 7.275	18.00	49.00	109

• **Std. Deviation**.

To conduct this study, Cone Beam Computed Tomography (CBCT) images that were first prepared for diagnostic reasons and collected from patients referred to the Department of Oral and Maxillofacial Radiology between 2019 and 2020 were carefully selected and examined.

Samples were primarily sourced from the right side due to its lower risk of trauma during intubation during infancy in hospitals, often performed by right-handed technicians.

To ensure a more precise evaluation of our sample, we stratified the age range from 18 to 49 years into three distinct groups:18–27 years, 28–37 years, and 38–49 years.the age group of 28–37 years constituted the largest proportion of the study. The breakdown of patient distribution by gender within each age group is provided in Table 2.

**Table 2:** Number of people studied in each age group by gender.

Age Groups (Years old)	Gender		Total Number (%)
	Male Number (%)	Female Number (%)	
**18–27**	12 (42.9)	16 (57.1)	28 (100)
**28–37**	23 (45.1)	28 (54.9)	51(100)
**38–49**	9 (30)	21 (70)	30 (100)
**Total**	44 (40.4)	65 (59.6)	109 (100)

The inclusion criteria were CBCT images of the maxillary central and first molar teeth in the age range of 19–49 years. These specific teeth were chosen because of their relevance in dental development and comparatively lower susceptibility to extraneous factors that could compromise the accuracy of age estimation. Potential confounding variables, such as caries, restoration, crown, pulpo-periapical lesion, severe attriton, or any major tooth damage, were considered exclusive. Images that did not meet the quality and resolution standards required for accurate analysis were removed to ensure data integrity.

The CBCT images were captured with the Planmeca ProMax 3D Classic imaging equipment from Helsinki, Finland. The Field of View (FOV) was set to 8 × 8, with an output range of 54–84 KVP and voxel sizes of .15 mm^3^.The samples were analyzed using the Planmeca Romexis Viewer v.3.8.3.R software.

Notably, this software was used to delineate the desired tooth borders across the different planes. The examination of CBCT images began with the observation of the axial planes. All of CBCT evaluation was performed by H.KH. The axial planes from the tip of the crown to the cementoenamel junction (CEJ) of the tooth were examined by moving the scroll bar. In cases of doubt, other plans were used to determine the CEJ position.

The sections of the desired tooth were carefully selected by moving the scroll bar. Subsequently, the isolated image of the desired tooth was saved in DICOM format and transferred to the ITK-SNAP 3.4.0 software (University of Pennsylvania, USA) environment for further analysis.

**Fig. 2:**
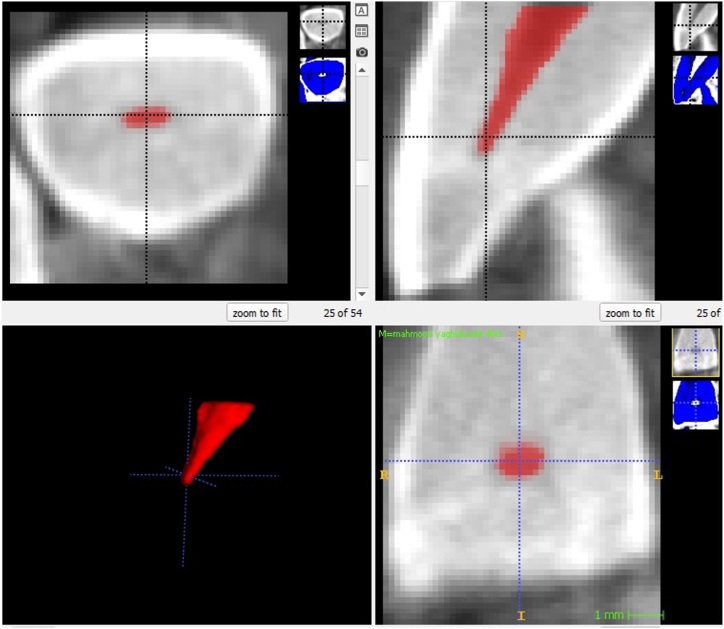

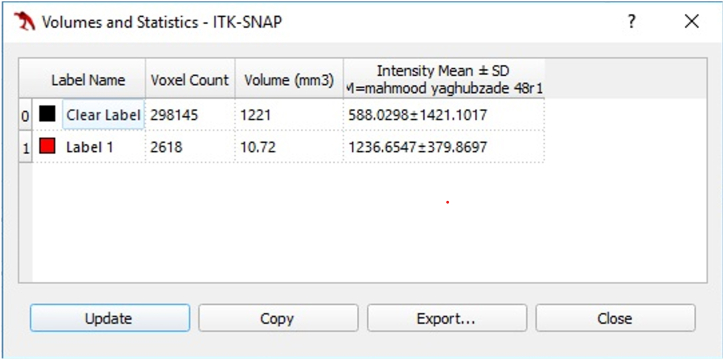
Examples of two volumetric images show how ITK-SNAP calculates the pulp and crown volumes based on the resolution contrast, which aids the red growing pattern in defining the boundaries of the pulp and surrounding dentin. a) When the red growth pattern is complete, the software calculates the volume of the designated region. b) By comparing the contrast between the pulp and crown areas, the software identifies the blue area and records its volume.

ITK-SNAP offers robust tools for semi-automatic segmentation, incorporating active contouring methods along with manual drawing and image-scrolling capabilities. This software is instrumental for cropping and measuring the selected portion of the tooth based on distinctive growth patterns [Fig. 2a and b].

Finally, within the ITK-SNAP software, the "Segmentation" category within the upper toolbar was selected, followed by the "Volumes and Statistics" menu. This process facilitated the quantification of the desired volume in cubic millimeters.

The data was then analyzed using PASW Statistics 18 (IBM Corp., Armonk, NY, USA) software. Descriptive statistics, and an independent *t*-test was used to compare the mean age between genders, if relevant. Linear regression analysis was used to develop age estimation methods, providing a mathematical foundation for the gathered data. All statistical tests in the study used a p-value threshold of <.05 to indicate statistical significance.

These extensive approaches, as indicated in Fig. 2a and b were critical to the study's success and helped to generate useful data for age estimation in the Iranian population.

## 3 Results

This cross-sectional study evaluated Cone-Beam Computed Tomography (CBCT) images from 109 patients. Table 1 presents descriptive data. Statistical analysis with an independent *t*-test found no statistically significant difference in mean age between male and female individuals (p = 0.46). The main aim of this research was to develop a mathematical model for estimating chronological age. The pulp-to-crown volume ratio, gender, and tooth type were all considered as factors influencing the study's outcome.

To achieve this goal, linear regression analysis was utilized and the following formula is the outcome:

Age = 42.04 – (3.28 × Tooth) – 144.60 × (Pulp to Crown Ratio)

In this equation, the variable "Tooth" assumes a value of one for the central incisor tooth and a value of two for the first molar tooth.

Additionally, our analysis of Table ІІІ revealed that male teeth exhibited a higher average pulp chamber and crown volume (mm^3^) compared to female teeth.

Specifically, when evaluating the maxillary central incisor and first molar teeth, with respective p-values of .261 and .260 in male and female, we observed that the average ratio of pulp to crown volume remained consistent across genders and for each tooth type. (Table 3) (see Table 4).

**Table 3:** Comparison of volumes of different dental components by gender and type of tooth.

Gender	Tooth	Side		Coronal pulp	Crown Volume	Pulp to Crown Ratio	P-value
**Male**	Central Incisor	Right	N	25	25	25	
			Mean	12.0323	364.6609	.0334320	
			Std. Deviation	5.08308	51.84831	.01427664	
		Left	N	3	3	3	
			Mean	18.4033	402.2887	.0454220	
			Std. Deviation	7.49551	39.61480	.01699486	
		Total	N	28	28	28	
			Mean	12.7149	368.6924	.0347167	
			Std. Deviation	5.58170	51.44182	.01472521	
	Maxillary First Molar	Right	N	14	14	14	.261
			Mean	16.3407	573.0872	.0285202	
			Std. Deviation	8.90432	148.98670	.01599722	

		Left	N	2	2	2	
			Mean	26.8125	749.3059	.0355515	
			Std. Deviation	6.58041	57.15622	.00607017	
		Total	N	16	16	16	
			Mean	17.6497	595.1146	.0293991	
			Std. Deviation	9.18670	151.91470	.01516622	
	Central Incisor	Right	N	36	36	36	
**Female**			Mean	12.1636	326.2461	.0367895	
			Std. Deviation	7.46779	85.90034	.01931092	
		Left	N	3	3	3	
			Mean	16.6080	403.3380	.0437362	
			Std. Deviation	8.12646	91.31016	.02535538	
		Total	N	39	39	39	
			Mean	12.5054	332.1762	.0373238	
			Std. Deviation	7.50203	87.56854	.01951472	
	Maxillary First Molar	Right	N	19	19	19	.260
			Mean	12.8485	489.5950	.0265291	
			Std. Deviation	8.15837	136.15204	.01266587	
		Left	N	7	7	7	
			Mean	25.2899	577.5717	.0456151	
			Std. Deviation	13.70284	126.03665	.02913940	
			N	26	26	26	
		Total	Mean	16.1981	513.2810	.0316676	
			Std. Deviation	11.16507	136.90534	.01984508	
	Central Incisor	Right	N	61	61	61	
**Total**			Mean	12.1098	341.9898	.0354135	
			Std. Deviation	6.54757	75.77939	.01737333	
		Left	N	6	6	6	
			Mean	17.5057	402.8133	.0445791	
			Std. Deviation	7.06087	62.95301	.01932720	
		Total	N	67	67	67	
			Mean	12.5930	347.4367	.0362343	
			Std. Deviation	6.72013	76.33414	.01759673	
	Maxillary First Molar	Right	N	33	33	33	
			Mean	14.3300	525.0159	.0273738	
			Std. Deviation	8.52771	145.60481	.01397144	
		Left	N	9	9	9	
			Mean	25.6282	615.7349	.0433788	
			Std. Deviation	12.11154	134.37620	.02571239	
		Total	N	42	42	42	
			Mean	16.7511	544.4557	.0308034	
			Std. Deviation	10.36325	146.59364	.01804254	
	Total	Right	N	94	94	94	
			Mean	12.8892	406.2437	.0325910	
			Std. Deviation	7.33595	136.79681	.01663672	
		Left	N	15	15	15	.123
			Mean	22.3792	530.5663	.0438589	
			Std. Deviation	10.89006	152.94239	.02261780	
		Total	N	109	109	109	
			Mean	14.1952	423.3523	.0341417	
			Std. Deviation	8.51480	144.90607	.01788463

**Table 4:** The results of other similar studies.

Author	Year	Tooth type	Age range	population	R^2^	Software	Gender Effect
**Ge**^**20**^	2016	UI	12–69	China	.323	ITK-SNAP	+∗
**Kumar**^**1**^	2016	UM1	10–70	India	.489	ITK-SNAP	+
**Biuki**^**15**^	2017	U1, U3	13–70	Iran	.58-.85	Mimics	+
**Asif**^**8**^	2018	U1	16–65	Malaysia	.78	Mimics	–
**Gulsahi**^**22**^	2018	U1	15<	Turkey	.53	3D-DOCTOR	–
**Hidayat**^**9**^	2018	U3, L3	13–73	Indonesia	.75	ITK-SNAP	–
**Zhang**^**18**^	2019	L8	20–65	China	.419	3D-SLICER	∗

∗ Positive (+) shows correlation between gender and the relation while Negative (−) doesn't. U1, Upper Central Incisor; UM1,Upper First Molar; U3, Upper Canine; L3,Lower Canine; L8,Lower Third Molar.

**Fig. 3:**
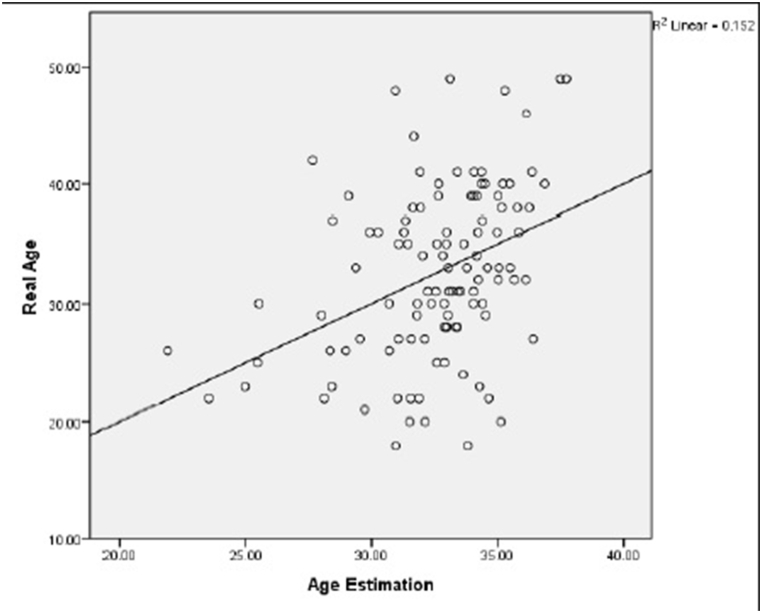

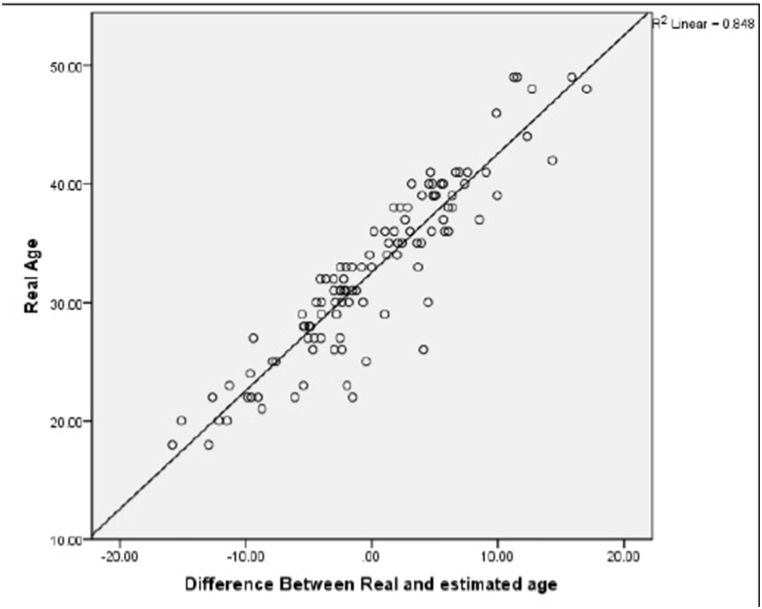

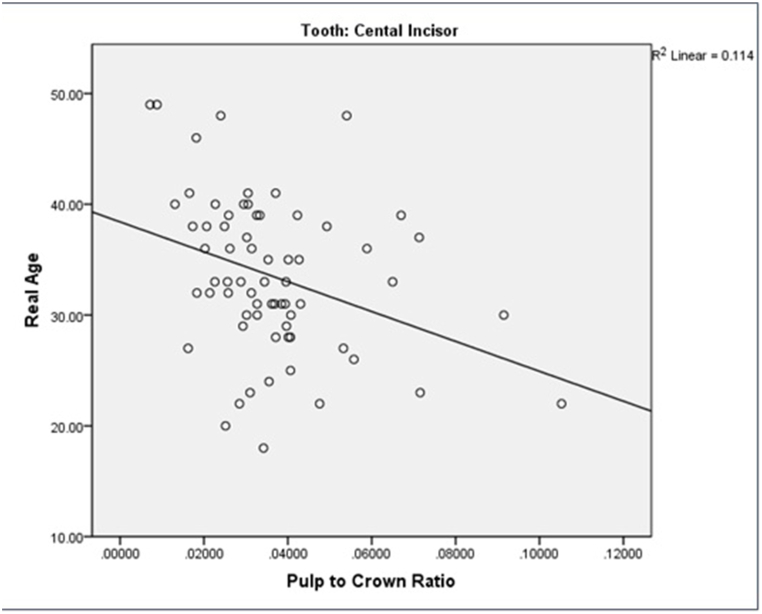

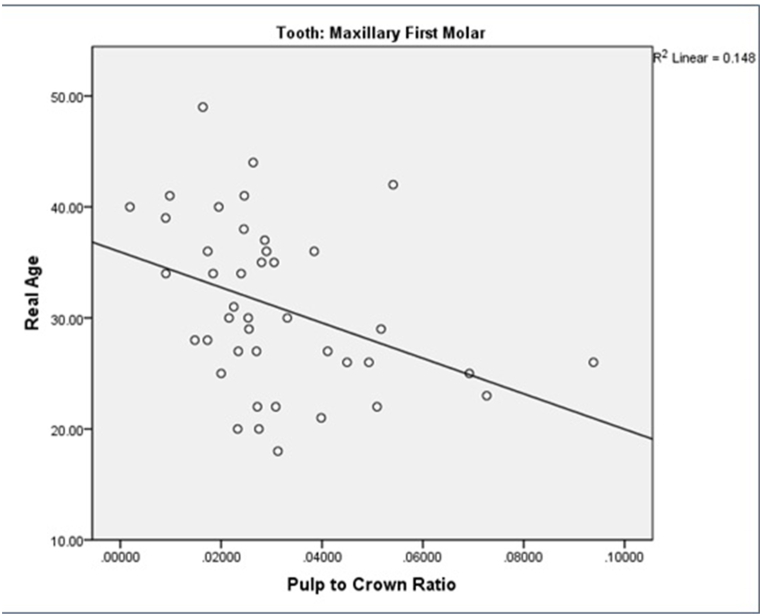
a) the relationship between actual and estimated age of the subjects. b) the relationship between “actual age” and “the difference between estimated and actual age” of the subjects. c) comparing the “actual age” with “the ratio of pulp chamber volume to crown volume” in maxillary central incisor. d) comparing the “actual age” with “the ratio of pulp chamber volume to crown volume” in first molar teeth.

Fig. 3a depicts the correlation between actual and estimated ages, revealing a moderate relationship. Especially, the actual age of the study participants ranged up to a maximum of 49 years, while the scatter chart illustrates a maximum estimated age of 38 years.

Fig. 3b provides additional insights, highlighting that the most accurate age estimates, falling within a range of ±5 years, are particularly applicable to individuals aged 28–37 years.

As shown in Fig. 3c and d, both tooth types exhibited a declining ratio of pulp-to-crown volume with advancing age.

The results of this study greatly enhance our knowledge of age estimate techniques and emphasize the practical use of the developed regression model for accurately predicting chronological age, especially within the age range covered by the study.

## 4 Discussion

Based on the pulp chamber to crown volume ratio of the maxillary central incisors and first molars, the age estimation approach works well for people in the 28–37 age range, with a 5-year interval; gender has no discernible effect on this connection.

In general, the reduction in pulp chamber volume due to secondary dentin deposition can be an indicator of age. Although two-dimensional images such as panoramic radiographs and periapical radiographs have been used to a limited extent for three-dimensional analysis of pulp decrease, the use of CBCT images to show morphological changes seems to be the most appropriate type of Imaging in dental age estimation [8,11,12]. This innovative approach affects the capabilities of CBCT to enhance the accuracy and effectiveness of age estimation, ultimately contributing to the advancement of forensic science. The development of such models represents a critical step forward in improving the precision and reliability of age estimation techniques, thereby supporting the endeavors of forensic experts and researchers worldwide [9].

Unlike some studies that used pulp volume for mandibular molar teeth, canine, impacted third molars; to estimate age, this study used a ratio of pulp chamber volume to the crown volume of the maxillary central incisors and first molars [[13], [14], [15]]. Central incisors and first molars are the first teeth to grow in the oral cavity, it seems that the reduction in pulp volume of these two types of teeth is a more appropriate indicator for age determination. Some studies have suggested that in teeth with larger pulp spaces, such as central incisors or canines, secondary dentin deposition can be detected better than in teeth with small pulp spaces, such as lateral incisors [16]. In addition, the process of secondary dentin production, followed by a reduction in pulp volume, is affected by occlusal forces. So the use of impacted tooth pulp volume does not seem reliable to determine age because it has not yet been subjected to any occlusal forces [13,15].

The end parts of roots and root canals are very small, measuring pulp volume in these areas is difficult and time-consuming, and the probability of measurement error is high. This is even more difficult in multirooted teeth. However, processes such as chewing and attrition over time cause secondary dentin deposition and a reduction in the volume of the crown and pulp of the tooth, while having no significant effect on the volume of the tooth root. Therefore, using the ratio of pulp chamber volume to crown volume to determine age may be preferable to other methods and has less error [17]. This was the reason for using the ratio of pulp chamber volume to crown volume as an age estimation index in the present study. Some studies used only pulp volume or pulp chamber volume to estimate the age but Gulsahi et al., like the present study, used the ratio of pulp chamber volume to tooth crown volume. This ratio compensates for the errors that occur due to anatomical differences among different people [14,[18], [19], [20]].

In a study by Star et al., the ratio of pulp to tooth volume was calculated.^(2)^ In the present study, the coefficient of determination (R^2^) for the maxillary central incisor teeth (R^2^ = .114) and maxillary first molar teeth (R^2^ = .148), indicated a weak-to-moderate correlation between the actual and estimated ages. Although the correlation between chronological age and the ratio of pulp chamber to crown volume was slightly stronger for the maxillary first molars, there was not much difference between the two types of teeth. In addition, gender did not play an important role in this study. The highest correlation was observed in the age range of 28–37 years with a difference of ±5 years between the estimated and actual ages.

In a study by Kumar et al., 27 % of the age differences in an Indian population aged 21–31 was explained by the pulp volume of the maxillary first molar. In men between the ages of 41 and 50, the maxillary first molar pulp chamber volume exhibited the strongest relationship with chronological age [1].

Asif et al. conducted a study in Egypt, revealing a significant correlation between chronological age and pulp-to-tooth volume ratio, pulp chamber-to-crown volume ratio in maxillary central incisor teeth and gender-independent mean pulp-to-tooth volume ratio in maxillary central incisors, as well as in the pulp chamber-to-crown ratio [8]. In Table ІV, the conditions and results of different similar studies are reported.

Contradictory results have been obtained by different investigations in this field, as is evident. The variations as mentioned earlier in secondary dentin secretion can be attributed to heredity, dietary patterns, and distinct behavioral patterns throughout the population. An increase in occlusal pressure from habits like clenching and bruxism can cause the pulp chamber volume to decrease and increase the production of secondary dentin.

Another reason for the difference between the results of the present study and some other studies is that the age range in our study was limited. Because it is possible to estimate the age according to the developmental stage of the tooth before the age of 18 years, in this study, the age range of the subjects was higher than 18 years. However, because it was difficult to find eligible teeth (no severe abrasion, calcification, caries, restoration and root canal therapy) the end of the age range was 49 years. However, in the study of Kumar et al., the age range of the study was 10–70 years and in the study of Hidayat et al., was 13–73 years [1,10].

Due to the fact that the pulp volumetric process was performed semi-automatically by the ITK-SNAP software with operator intervention, this process can be considered as a factor for the difference in results. This factor is also considered in the study of Hidayat et al. [10] The pulp volumetry process in our study was semi-automated using ITK-SNAP software, involving operator intervention. This aspect could potentially introduce variability into the results, an aspect also considered in Hidayat et al.'s study [10]. Furthermore, the accuracy of the different software used across studies may have influenced the results.

Different studies may have different results due to sample size, CBCT device type, type of teeth examined, and methods used. Variations and approaches used in each study can also contribute to the disparity in results. For example, analyzing pulp volume, pulp chamber volume, pulp-to-tooth volume ratio, or pulp chamber-to-crown volume ratio may be different approaches used in each study.

The study suggests using larger sample sizes and exploring other teeth, such as canines with larger pulp chambers and lower susceptibility to decay or loss, could be advantageous for age estimation research. CBCT images are preferred for comprehensive three-dimensional evaluation of pulp chamber changes in dental age estimation [8,11,12]. The study focused on the ratio of pulp chamber volume to crown volume in maxillary central incisors and first molars, Pulp volume estimation in impacted teeth is less reliable due to their minimal exposure to occlusal forces.^(16)^Measuring pulp volume in roots and root canals is challenging due to their diminutive dimensions. However, processes like chewing and attrition over time lead to secondary dentin deposition and a reduction in crown and pulp volumes while having a negligible impact on tooth root volume. The ratio of pulp chamber volume to crown volume is a preferable approach with reduced susceptibility to error [17].

## 5 Conclusions

The study estimates the age in the Iranian population using CBCT of maxillary central incisors and first molars. **The pulp chamber to crown volume ratio in maxillary central incisors and first molars is a reliable age indicator for 28–37 years, with a ±5 year difference, but its accuracy decreases when applied to other age groups.** Gender does not significantly influence the age regression equation or estimation process. larger sample sizes and exploring other teeth, such as canines with larger pulp chambers and lower susceptibility to decay or loss, could be advantageous for age estimation research.

## CRediT authorship contribution statement

**Atie Safaei:** Writing – review & editing, Methodology, Conceptualization. **Ali Bagherpour:** Writing – review & editing, Formal analysis. **Shahrokh Naseri:** Formal analysis. **Mahsa Etemadi:** Writing – review & editing, Writing – original draft, Conceptualization, Investigation, Methodology, Resources. **Hossein Khoshkhou:** Writing – review & editing, Writing – original draft, Resources, Investigation, Conceptualization, Methodology.

## Conflict of interest

The authors of this manuscript declare that they have no conflicts of interest, real or perceived, financial or non-financial in this article.

## Data availability statement

Data will be made available on request.

## Funding

This research did not receive any specific grant from funding agencies in the public, commercial, or not-for-profit sectors.

## Declaration of competing interest

The authors declare that they have no known competing financial interests or personal relationships that could have appeared to influence the work reported in this paper.
